# Maximizing the Value of Mobile Health Monitoring by Avoiding Redundant Patient Reports: Prediction of Depression-Related Symptoms and Adherence Problems in Automated Health Assessment Services

**DOI:** 10.2196/jmir.2582

**Published:** 2013-07-05

**Authors:** John D Piette, Jeremy B Sussman, Paul N Pfeiffer, Maria J Silveira, Satinder Singh, Mariel S Lavieri

**Affiliations:** ^1^VA Center for Clinical Management Research and Division of General MedicineDepartment of Internal MedicineUniversity of MichiganAnn Arbor, MIUnited States; ^2^VA Center for Clinical Management Research and Department of PsychiatryAnn Arbor VA Healthcare System and University of MichiganAnn Arbor, MIUnited States; ^3^Artificial Intelligence LaboratoryDepartment of Electrical Engineering and Computer Science, College of EngineeringUniversity of MichiganAnn Arbor, MIUnited States; ^4^Deparment of Industrial and Operations EngineeringCollege of EngineeringUniversity of MichiganAnn Arbor, MIUnited States

**Keywords:** cellular phone, telemedicine, depression, self-care

## Abstract

**Background:**

Interactive voice response (IVR) calls enhance health systems’ ability to identify health risk factors, thereby enabling targeted clinical follow-up. However, redundant assessments may increase patient dropout and represent a lost opportunity to collect more clinically useful data.

**Objective:**

We determined the extent to which previous IVR assessments predicted subsequent responses among patients with depression diagnoses, potentially obviating the need to repeatedly collect the same information. We also evaluated whether frequent (ie, weekly) IVR assessment attempts were significantly more predictive of patients’ subsequent reports than information collected biweekly or monthly.

**Methods:**

Using data from 1050 IVR assessments for 208 patients with depression diagnoses, we examined the predictability of four IVR-reported outcomes: moderate/severe depressive symptoms (score ≥10 on the PHQ-9), fair/poor general health, poor antidepressant adherence, and days in bed due to poor mental health. We used logistic models with training and test samples to predict patients’ IVR responses based on their five most recent weekly, biweekly, and monthly assessment attempts. The marginal benefit of more frequent assessments was evaluated based on Receiver Operator Characteristic (ROC) curves and statistical comparisons of the area under the curves (AUC).

**Results:**

Patients’ reports about their depressive symptoms and perceived health status were highly predictable based on prior assessment responses. For models predicting moderate/severe depression, the AUC was 0.91 (95% CI 0.89-0.93) when assuming weekly assessment attempts and only slightly less when assuming biweekly assessments (AUC: 0.89; CI 0.87-0.91) or monthly attempts (AUC: 0.89; CI 0.86-0.91). The AUC for models predicting reports of fair/poor health status was similar when weekly assessments were compared with those occurring biweekly (*P* value for the difference=.11) or monthly (*P*=.81). Reports of medication adherence problems and days in bed were somewhat less predictable but also showed small differences between assessments attempted weekly, biweekly, and monthly.

**Conclusions:**

The technical feasibility of gathering high frequency health data via IVR may in some instances exceed the clinical benefit of doing so. Predictive analytics could make data gathering more efficient with negligible loss in effectiveness. In particular, weekly or biweekly depressive symptom reports may provide little marginal information regarding how the person is doing relative to collecting that information monthly. The next generation of automated health assessment services should use data mining techniques to avoid redundant assessments and should gather data at the frequency that maximizes the value of the information collected.

## Introduction

Clinicians and health care payers increasingly look to mobile health services such as Interactive Voice Response (IVR) as tools for monitoring patients’ status between face-to-face encounters and identifying individuals who need attention to prevent acute events [1-3]. Multiple studies have shown that IVR monitoring yields actionable and reliable clinical information even on sensitive topics such as mental health and substance abuse [4-11]. Moreover, patients are willing to complete regular IVR assessments over extended periods of time, even when challenged by chronic illness, age, poverty, low literacy, and psychiatric problems [12,13].

While IVR has significant potential to increase the information base of proactive care management, the design of automated monitoring services can have negative consequences that should be carefully considered when deciding the frequency and content of each assessment call. Studies suggest that patients may tire of frequent IVR assessments [12-15], particularly if they are asked repeatedly for information about health or self-care problems that have not changed. At the same time, many patients have a large number of health problems associated with multiple chronic conditions [16,17]. For such patients, current alternatives to the typical disease-specific focus include substantially increasing the length of each assessment, increasing the frequency of assessment calls, focusing on a broader number of problems but with less depth on each, or focusing only on cross-cutting issues such as medication adherence or physical activity. Each of these strategies introduces new challenges to sustaining patient engagement or the quality of information for clinical decisions. As with other types of patient contact [18-21], the timing and content of IVR monitoring is almost always based on expert opinion and static flow diagrams. As such, these systems have not achieved their full potential as a strategy for cost-effectively increasing patients’ access to between-visit monitoring and self-care support.

While frequent (eg, weekly or daily) IVR assessment calls may be necessary to detect fluctuations in important health indicators, what if a patient’s IVR assessment reports could be predicted based on the information that he or she provided in prior calls? For example, if a patient has consistently reported perfect medication adherence over multiple prior IVR assessments, what would be the probability that they would report something different today? Data mining is a set of analytic techniques designed to extract latent information from data in order to make predictions about the future [22,23]. In the context of IVR, data mining could help identify when patients’ answers are so stable that the same questions are not worth asking again, or when there are changes in the patient’s status indicating the need for more intensive probing. Using information about such patterns, adaptive mobile health monitoring programs could be developed that automatically adjust the frequency and content of assessments so that they provide the most useful information for guiding patient counseling and clinical follow-up.

We used one approach to data mining in order to examine data from 1050 IVR assessments of 208 patients with depression diagnoses. All patients received IVR calls at regular intervals, during which they completed the Patient Health Questionnaire (PHQ-9) [24,25], a widely used and validated depression assessment scale. Also, patients repeatedly answered questions regarding their antidepressant medication adherence, perceived general health, and days in bed due mental health problems. Given the large number of serial reports from each patient, we examined the predictability of patients’ IVR responses. Specifically, for each patient we identified the five most recent weekly, biweekly, and monthly assessments. We used those data plus other information collected during prior assessments and at the time of the patient’s enrollment to determine the extent to which health reports were predictable and whether that predictability varied according to the frequency of attempted assessment calls. Based on these analyses, we determined whether less frequently collected data (eg, biweekly or monthly) provided as much information about patients’ status as information collected weekly, thereby making it possible to decrease the frequency of IVR calls or to change their focus to other important health indicators. More generally, we sought to determine whether data mining techniques might inform automated assessments that repeatedly measure patients’ health status, so that the most clinically useful, nonredundant information is collected.

## Methods

### Patient Eligibility and Recruitment

Patients were enrolled between March 2010 and January 2012 from 13 university-affiliated and community-based primary care practices. To be eligible, patients had to have two primary care visits in the previous 2 years, at least one in the previous 13 months, and either a depression diagnosis listed in clinical records or an antidepressant prescription plus a diagnosis of depression listed in billing data. Patients with schizophrenia, psychosis, delusional disorder, bipolar disorder, or dementia were excluded. Potential participants were mailed an introductory letter that was followed by a screening and recruitment telephone call. Patients who provided informed consent were enrolled in the IVR system and mailed additional program information, including materials describing effective communication with informal caregivers and clinicians. The study was approved by the human subjects committees of the University of Michigan and Ann Arbor VA Healthcare System. More information about the intervention and patients’ engagement in the IVR calls has been published elsewhere [13].

### IVR Monitoring Protocol

Detailed information about the IVR call content and functioning are available by contacting the authors. In brief, each week that an assessment was scheduled, the system made up to three attempts to contact the patient on up to three different patient-selected day/time combinations. The content of the calls was developed with input from psychiatrists, primary care providers, and experts in IVR program design and health behavior change. Every call included an assessment of patients’ depression symptoms using the PHQ-9 [24]. The PHQ-9 is a 9-item questionnaire that is sensitive and specific with respect to other established measures of major depression. Scores are associated with physical functioning, sick days, and health care use [24]. Because self-rated health status is correlated with patients’ service use and mortality risk [26-28], they were asked the standard item, “Thinking about your overall health, how were you feeling this past week (excellent, very good, good, fair, poor)?” Medication adherence was assessed by asking: “How often during the past week did you take your depression medication exactly as prescribed (always, most of the time, less than half of the time, rarely or never)?” Finally, during each assessment, patients were asked: “This past week, did you ever stay in bed all or most of the day because of your mental health (yes versus no)?” Calls used tree-structured algorithms to present recorded queries and tailored information that was invariant across patients and over time. Based on patients’ responses, they received tailored advice for managing their self-care. For example, patients’ received messages tailored according to their recent trajectories in depression scores (trending positive, negative, or stable and by how much), including messages such as the following:

It sounds like you’re still experiencing some serious symptoms of depression. Remember that if you’re prescribed a medication for depression, it’s important that you keep taking it exactly as prescribed to keep your depression from getting worse. Sometimes it takes awhile for a depression medication to work, so if you have been on your current medication for less than 8 weeks, try to be patient and see if you start to see some improvement. If you’ve been on the same medication for more than 8 weeks and you’re still not feeling okay, your doctor wants to know. You should make an appointment with your doctor to talk about some other treatment options. I’ll give you the phone number of your doctor’s office at the end of this call.

Clinicians received fax alerts identifying patients reporting health problems requiring follow-up before their next outpatient encounter. For patients enrolling with a family caregiver, those caregivers received automatic updates by IVR and email with suggestions regarding how they could support the patient’s self-management.

### Outcomes of Interest

For each assessment, we created binary indicators for each of the four outcomes reported: (1) moderate/severe depressive symptoms indicated by a PHQ-9 score of ≥10; (2) fair or poor perceived general health status; (3) poor antidepressant adherence, ie, rarely or never taking antidepressant medication as prescribed; and (4) spending days in bed in the past week due to mental health problems.

### Analytic Sample Definition and Analyses

In order to determine the predictability of patients’ assessment reports based on the content and frequency of prior assessments, we identified the subset of patients with one or more “index” assessments meeting the following criteria: (a) five completed prior assessments immediately preceding the index assessment and collected with the program’s normal frequency of weekly assessment attempts; (b) five completed prior assessments with a 2-week minimum gap between each one; and (c) five completed prior assessments with a minimum 4-week gap between each one. A total of 1050 index assessments for 208 unique patients were identified.

In addition to linking each index assessment to prior assessment information, index assessments also were linked with information about that patient’s sociodemographic and clinical characteristics collected at the time of program initiation. Those baseline data included patients’ age, gender, educational attainment, baseline depressive symptom severity score (ie, measured using the PHQ-9 minus the item asking about suicidal ideation [29]), self-reported hospital admission in the year prior to program entry, physical functioning as measured by the SF-12 [30], and the number of comorbid chronic medication conditions.

In initial analyses, we examined the correlation across the four health indicators reported within each index assessment, and we calculated the alpha reliability of patients’ IVR-reported PHQ-9 scores. We then examined the proportion of patients reporting each health problem in the index assessment when the same problem was reported in the one or in both of the most recent prior assessments assuming weekly, biweekly, or monthly assessment attempts. For example, we examined measures of association between patient reports of moderate/severe depressive symptoms (PHQ-9 ≥10) and similarly high PHQ-9 scores in the most recent assessment or both of the two most recent assessments (assuming weekly, biweekly, and monthly assessment calls).

Finally, we fit multivariate logistic regression models predicting each of the four health indicators as reported in index assessments. Each model included patients’ baseline sociodemographic and clinical characteristics as defined above, as well as information about that same health indicator and the other three health indicators reported in five prior assessments collected assuming a periodicity of weekly, biweekly, or monthly call attempts. Serial indicators designed to capture additional information about trends in patients’ depression scores (eg, the number of weeks since program entry and prior number of completed assessments) also were considered as potential predictors. For models predicting moderate/severe depressive symptoms, fair/poor health, and days in bed, these additional variables had no discernible marginal predictive value in the context of the multiple prior, ordered indicators of the patient’s health and self-care. However, an indicator for weeks since program entry was a marginally significant predictor of patients’ medication adherence and was retained in the models used as the basis of ROC curves predicting patient reports of poor antidepressant medication adherence.

When fitting each of the three models, we used two strategies to prevent overfitting to the current dataset. First, we used 10-fold cross validation, in which the model was fit 10 times based on random 90% training samples and then used to predict the outcomes in mutually exclusive 10% test samples. Second, for each of the ten replications, we used stepwise regression (with a *P* value of .20 for removal) to identify the most significant subset of candidate predictors. All models also adjusted for clustering of assessment responses by patient.

The predictive significance of the three models for each outcome was compared graphically to one another and to a model with only baseline information using Receiver Operator Characteristic (ROC) curves. We also compared the area under the curve (AUC) across ROCs and calculated each AUC’s 95% confidence interval [31]. To illustrate the potential predictive accuracy of the best model for each outcome, we report the sensitivity and specificity at the point on the ROC curve with the highest proportion of outcomes correctly predicted.

## Results

### Patient Characteristics

Patients were on average 52.2 years of age. Most were women, white, and married (Table 1). Patients reported a mean of 2.4 comorbid chronic conditions including hypertension (50.0%), arthritis (49.5%), chronic lung disease (33.2%) and back pain (42.1%). Roughly a third (33.2%) of patients had moderate or severe depressive symptoms at baseline; those patients were somewhat younger on average at the time of program enrollment than patients with mild depressive symptoms.

### Co-Occurrence of Reported Health Problems Within IVR Assessments

Patients reporting a given problem during their IVR assessments were more likely to report other concurrent problems as well. For example, compared to patients reporting mild depressive symptoms, those reporting moderate/severe depressive symptoms were more likely also to report staying in bed all or most of the day due to mental health problems (27% versus 8%) and that their general health was either fair or poor (47% versus 14%, both *P*<.001 after adjusting for clustering by patient). Similarly, patients reporting being bedbound during the past week due to mental health problems were significantly more likely than other patients to rate their health as fair or poor during the same assessment (29% versus 20%, *P*<.001). Patients reporting that they rarely or never took their medication as prescribed were more likely than other patients to report poor general health (28% versus 17%; *P*<.001).

### Bivariate Relationship Between IVR Reports and Prior Reports of the Same Outcome

The internal reliability of the PHQ-9 was excellent (alpha=.87). Patients were substantially more likely to report moderate/severe depressive symptoms if they reported similar information in prior assessments (Table 2). For example, while patients reported moderate/severe depressive symptoms in 21.5% of all assessments, they did so 70.3% of the time when they also reported similarly high symptoms on their most recent assessment, and 83.3% of the time when they reported moderate/severe depressive symptoms during both of their most recent assessments, assuming weekly assessment attempts. Ninety-one percent of patients whose most recent weekly PHQ-9 score was <10 also had a score <10 on their index assessment. Assuming weekly assessment attempts, a similar pattern was observed with respect to the autocorrelation of patients’ reported general health status, medication adherence, and days in bed due to mental health problems.

In general, assessments collected biweekly or monthly were only somewhat less correlated with subsequent reports than information collected assuming weekly assessment attempts. For example, 58.8% of index assessments in which the patient reported moderate/severe depressive symptoms had similarly high levels in the two most recent assessments collected assuming weekly attempts, as compared to 53.4% on the two prior assessments collected biweekly, and 51% on the two prior assessments collected monthly.

### Predictive Models

#### Moderate/Severe Depression

**Figure 1:**
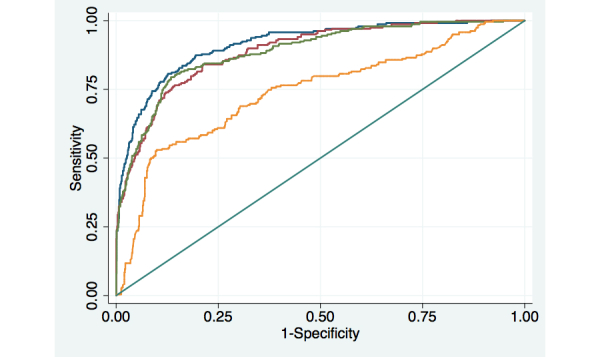
Receiver Operator Characteristic (ROC) curves for models predicting patient reports of moderate/severe depression, as measured by a PHQ-9 score ≥10.

ROC curves for models predicting patients’ depressive symptoms were highly predictive with an AUC≥0.89 regardless of whether prior assessments were attempted weekly, biweekly, or monthly (Figure 1 and Table 3). In Figure 1, the blue line represents weekly assessment attempts, the green line represents biweekly attempts, and the red line represents monthly attempts. The yellow line represents the ROC curve for the model predicting depressive symptoms using baseline data only. All other models also included baseline clinical and sociodemographic information. While the AUC for weekly assessments was significantly different than either biweekly (*P*<.001) or monthly assessments (*P*<.001), there was no statistically significant difference in the AUC for biweekly compared to monthly calls (*P*=.36).

The AUC for the model assuming weekly assessment attempts was .91 (95% CI 0.89, 0.93). At the point on the ROC curve with the greatest number of reports correctly classified (ie, a probability of moderate/severe depression=.50), 88.4% of assessments were classified correctly with a sensitivity of .68 and a specificity of .94. As expected, regardless of the frequency of assessment attempts, patients’ prior PHQ-9 scores were the strongest predictor of index assessment scores ≥10, although prior IVR reports regarding general health status, baseline depressive symptom severity, baseline physical functioning, and the number of comorbidities reported at baseline also were significant independent predictors of patients’ depression status.

#### General Health Status

**Figure 2:**
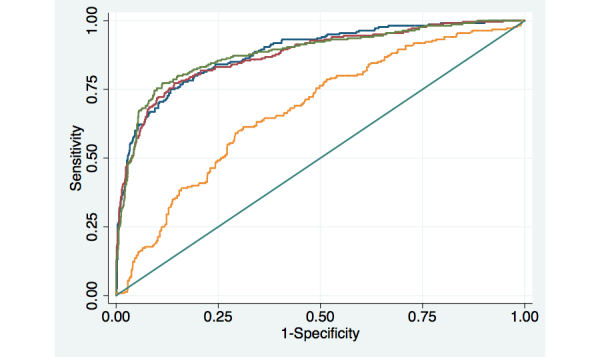
Receiver Operator Characteristic (ROC) curves for models predicting patient reports of fair or poor general health status.

Similar to patients’ reports of their depressive symptoms, reports of perceived general health status were highly predictable based on prior information (Figure 2). In Figure 2, the blue line represents weekly assessment attempts, the green line represents biweekly attempts, and the red line represents monthly assessment attempts. The yellow line represents the prediction based on baseline data only. All other models also included baseline clinical and sociodemographic information.

The AUC for the model assuming weekly assessment attempts was 0.88 (95% CI 0.86, 0.91). The AUC for that model was not statistically different from the one assuming biweekly attempts (*P*=.11) or assessments collected monthly (*P*=.81). Prior reports of perceived health status were the strongest predictors, although prior information about days in bed due to mental health problems and about medication adherence problems also were consistently retained in logistic models as predictors of patients’ index assessment reports of fair/poor health. With respect to the model assuming weekly assessment attempts, the cutoff indicating a probability of fair/poor health=.50 correctly classified 87% of all index assessments, with a sensitivity of .58 and a specificity of .95.

**Table 1:** Patient characteristics (cell entries, aside from N, are either column percent or mean [SD]).

	Depressive symptom severity^a^			
	Total	Moderate/Severe	Mild	*P* value
N	208	69	139	
Age in years	52.2 (12.5)	50.6 (12.0)	53.7 (12.8)	.04
Female	79.0	77.9	80.0	.72
White	90.0	89.5	90.5	.81
Married	60.0	57.9	62.1	.55
More than high school	79.5	75.8	83.2	.21
Prior hospitalization^b^	21.6	24.2	19.0	.38
Number of diagnoses	2.4 (1.7)	2.6 (1.7)	2.2 (1.6)	.09
Hypertension	50.0	55.8	44.2	.11
Cardiovascular disease	8.4	10.5	6.3	.30
Stroke	4.2	4.2	4.2	1.00
Arthritis	49.5	52.6	46.3	.38
Chronic lung disease	33.2	41.1	25.3	.02
Back pain	42.1	43.2	41.1	.77
Physical functioning^c^	39.6 (13.8)	37.8 (14.2)	41.4 (13.3)	.07

^a^PHQ-9: 9-item Patient Health Questionnaire score ≥10 or <10.

^b^1+ hospitalizations in the year prior to enrollment.

^c^Physical Functioning: 12-item Medical Outcome Study Short Form Physical Composite Summary. Scores range from 0 to 100 with higher scores indicating greater functioning.

**Table 2:** Variation in problem reports by the number and frequency of prior reports of the same problem.

		Weekly		Biweekly		Monthly	
		1 report^a^	2 reports^b^	1 report	2 reports	1 report	2 reports
**Moderate/Severe Depression** ^c^							
	% with Report^d^	21.5	21.5	21.5	21.5	21.5	21.5
	Sensitivity^e^	69.6	58.8	69.1	53.4	64.2	51.0
	Specificity^f^	92.0	96.8	90.5	95.4	89.8	95.8
	PPV^g^	70.3	83.3	66.5	76.2	63.3	77.0
	NPV^h^	91.7	89.6	91.5	88.2	90.2	87.7
**Fair/Poor Health**							
	% with Report	21.4	21.4	21.4	21.4	21.4	21.4
	Sensitivity	67.2	57.4	67.2	57.4	67.7	55.9
	Specificity	90.5	96.7	89.4	96.7	89.7	96.4
	PPV	65.9	82.4	63.4	82.4	64.2	80.9
	NPV	91.0	89.3	90.9	89.3	91.0	88.9
**Poor Adherence**							
	% with Report	18.6	18.6	18.6	18.6	18.6	18.6
	Sensitivity	55.5	43.2	54.2	42.6	58.4	39.6
	Specificity	90.4	96.2	91.2	96.6	90.1	96.5
	PPV	57.0	72.0	58.3	74.2	57.3	71.8
	NPV	89.9	88.1	89.7	88.1	90.5	87.5
**In Bed Due to Mental Health**							
	% with Report	12.9	12.9	12.9	12.9	12.9	12.9
	Sensitivity	45.4	24.2	39.5	17.5	30.3	11.7
	Specificity	91.8	97.3	91.0	97.3	90.7	97.4
	PPV	45.0	55.8	39.5	47.7	32.7	38.9
	NPV	91.9	90.0	91.0	89.2	89.7	88.6

^a^Patient also reported the same health problem in the most recent assessment during the time frame.

^b^Patient also reported the same health problem in the two most recent assessments during the time frame.

^c^PHQ-9 score ≥10.

^d^Percentage of all index assessments in which that health problem was reported.

^e^Proportion of index assessments reporting that health problem that also had the problem reported in the prior assessment(s).

^f^Proportion of index assessments not reporting that health problem that also were negative in the prior assessment(s).

^g^PPV: Positive Predictive Value; given that the problem was reported in the prior assessment(s), the proportion reporting that problem in the index assessment.

^h^NPV: Negative Predictive Value; given that the problem was not reported in the prior assessment(s), the proportion of index assessments that also did not report the problem.

**Table 3:** Area under the Receiver Operator Characteristic (ROC) curve for logistic models predicting each health indicator assuming different assessment frequencies.

		AUC^a^	95% CI
**Moderate/Severe Depression** ^b^			
	Weekly	0.9139	0.8931, 0.9348
	Biweekly	0.8887	0.8655, 0.9119
	Monthly	0.8873	0.8630, 0.9116
	Baseline data only	0.7396	0.7010, 0.7782
**Fair/Poor General Health**			
	Weekly	0.8840	0.8581, 0.9100
	Biweekly	0.8758	0.8477, 0.9039
	Monthly	0.8822	0.8543, 0.9101
	Baseline data only	0.6760	0.6367, 0.7154
**Poor Antidepressant Adherence**			
	Weekly	0.8396	0.8035, 0.8757
	Biweekly	0.8268	0.7899, 0.8637
	Monthly	0.8350	0.8000, 0.8701
	Baseline data only	0.7578	0.7162, 0.7993
**In Bed Due to Mental Health**			
	Weekly	0.7522	0.7058, 0.7986
	Biweekly	0.6872	0.6358, 0.7385
	Monthly	0.7197	0.6716, 0.7677
	Baseline data only	0.6029	0.5542, 0.6515

^a^Area Under the Curve.

^b^PHQ-9 score ≥10.

#### Poor Antidepressant Adherence

**Figure 3:**
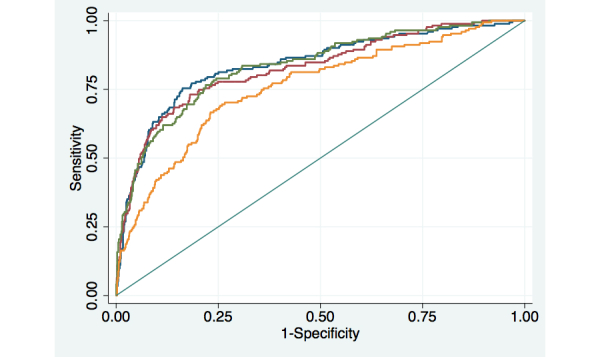
Receiver Operator Characteristic (ROC) curves for models predicting patient reports of poor antidepressant medication adherence.

While the overall predictive power was somewhat lower across models predicting reports of medication adherence problems, those models also showed that information collected biweekly or monthly was similar in its correlation with index assessment reports compared to information collected weekly (Table 3 and Figure 3). In Figure 3, the blue line represents weekly assessment attempts, the green line represents biweekly attempts, and the red line represents monthly attempts. The yellow line represents the ROC curve for the model predicting poor adherence using baseline data only. All other models also included baseline clinical and sociodemographic information.

The AUC for the model based on weekly assessments was 0.84 (95% CI 0.80, 0.88). The AUC for that model was not significantly different compared to either biweekly (*P*=.07) or monthly (*P*=.60) assessment attempts. In addition to prior information about patients’ medication adherence, patients’ age and baseline physical functioning consistently contributed to the predictive power of these models. Assuming weekly assessment attempts, the point on the ROC curve with the greatest number of assessments correctly classified (probability of adherence problems=.58) had a sensitivity of .86 and a specificity of .41.

#### Days in Bed

**Figure 4:**
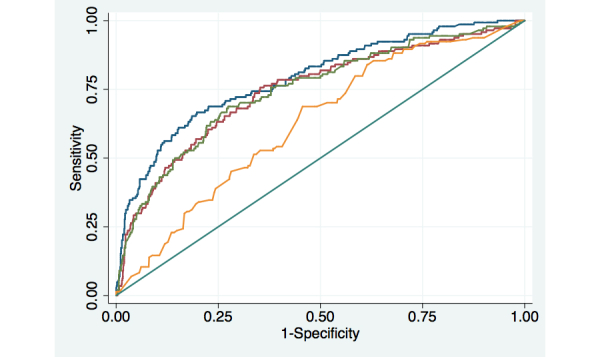
Receiver Operator Characteristic (ROC) curves for models predicting patient reports of being bedbound due to mental health problems.

Models predicting days in bed due to mental health problem had the lowest predictive accuracy as measured by the AUC’s for models based on weekly, biweekly, and monthly assessment attempts (Table 3 and Figure 4). In Figure 4, the blue line represents the ROC curve for the model based on weekly assessment attempts, the green line represents biweekly assessment attempts, and the red line represents monthly attempts. The yellow line represents the prediction with baseline data only, and all other models also included baseline clinical and sociodemographic information. While the AUC for weekly assessments was significantly different than either biweekly (*P*=.05) or monthly assessments (*P*=.05), there was no statistically significant difference in the AUC for biweekly and monthly calls, (*P*=.57). In addition to the patient’s prior reports of days in bed, prior reports of depressive symptoms, as well as their baseline physical and mental functioning were significant predictors of days in bed.

## Discussion

### Principal Findings

These analyses suggest that some IVR assessments of health and behavioral risk factors among patients with depression diagnoses may be unnecessary because patients’ responses are predictable based on their prior pattern of reports. In particular, we found that there is little to be gained from asking patients to report their PHQ-9 depression scores weekly and only a negligible incremental difference between biweekly and monthly assessment attempts. A similar pattern was observed with patients’ reports of fair or poor perceived general health.

Less frequent assessments of a given health indicator, particularly when that indicator is measured via a multi-item scale such as the PHQ-9, would have two benefits. First, it may be possible to decrease patients’ response burden and risk for dropout by avoiding repetitive assessments of the same health problem. Second, by avoiding redundancy in IVR monitoring, more efficient messages could be designed that would cover a broader range of clinical parameters. In the current study, patients reported an average of more than two comorbid chronic conditions. Minimizing redundant questioning would allow for more comprehensive monitoring of comorbidities that may complicate the treatment of patients’ depression and pose an independent threat to patients’ health.

For two of the outcomes we examined—medication nonadherence and bed-bound status—prior IVR reports were only moderately successful in predicting patients’ responses in a subsequent call. Several explanations are possible. It may be that adherence and days in bed were not reliably measured or that other still unmeasured predictors are more important in determining these health behaviors prospectively. Or it may be that these health indicators were in fact changing in unpredictable ways more rapidly than the frequency of monitoring could detect. If the latter reason is true, it may mean that even more frequent assessments are needed to detect all problems that arise. In any case, the approach to examining the frequency of monitoring presented here represents a framework for evaluating those options and making more informed choices about what health indicators to monitor and how often.

Assessments conducted in the current study were completed as part of a clinical service, with feedback to patients’ primary care team and informal caregivers when serious problems were reported. It may be that those feedback reports led to interventions that stabilized patients’ health status in ways that made subsequent patient reports more predictable. For obvious reasons, collecting patient health information without acting on it would be ethically challenging, but such information could provide insights into the appropriate periodicity of IVR monitoring for various outcomes. On the other hand, data used in the current study are more representative of what patients are likely to report in “real-world” practices, and the fact that we found that weekly assessments may produce redundant information is encouraging for health care organizations struggling with how best to manage their patients with multiple, competing health demands.

Patients who recently changed their antidepressant medication regimen may be more likely to experience side effects leading to adherence problems. The current system was not linked to pharmacy records. Such linkages represent an excellent example of the way in which monitoring systems that include a broader array of potential determinants of patients’ health may help ensure that mobile health services focus on health indicators providing the most prognostically important information in the context of everything that is known about the patient.

Predictive models such as these could be used along with advanced machine learning algorithms to tailor the frequency of monitoring across patients, time, and health indicators. For example, time saved gathering redundant information about the trajectory of patients’ depressive symptoms could be used to provide cognitive behavioral therapy designed to improve patients’ mood by teaching skills such as cognitive restructuring or increased pleasurable activities [32]. Or for patients with depression and comorbid medical disorders, more efficient algorithms could adapt automatically in order to focus on the patient’s other diseases, symptoms, or self-care behaviors that need greater attention to promote overall wellness. In brief, data mining approaches illustrated in the current study could be linked with algorithms that automatically update the content of patients’ repeated mobile health interactions, maximizing the emphasis on patient education while continually monitoring the health problems that pose the greatest risk to patients’ current and future risk for complications.

Each of the four outcomes examined could have been characterized using ordinal or even continuous measures, and the choice of dichotomizing the outcomes may have decreased the models’ predictive power. We chose binary outcomes because clinical decisions (eg, whether to call the patient, request a visit, or change a prescription) are often binary, and these logistic models lend themselves to comparison via ROC curves that are familiar to many health care professionals. Nevertheless, data mining includes an increasingly large armamentarium of approaches that could be brought to bear on clinical prediction problems, depending on (for example) the functional form of the outcome, the amount of data available, and whether the relationship of interest is represented by “noisy” data generated from an underlying parametric model.

The current study used logistic regression, cross validation, and ROC curves to identify the predictive trends in patients’ IVR-reported data. Artificial neural networks (ANNs) are an alternative parametric approach with more than 15 years of applications to medical diagnostics [33]. Support Vector Machines [34] represent a popular, nonparametric alternative to ANNs [35] for complex classification problems, particularly when the boundaries between groups (eg, between depressed and nondepressed patients) are irregular with respect to predictor variables and sufficient data are available for classification despite noise. Hierarchical latent-variable models (eg, Hidden-Markov Models [36]) could be used to capture underlying latent determinants of depression scores so that medical decisions can be conditioned on that latent information. If a continuous depression score were the outcome, moving average models with exponential smoothing could provide an initial understanding of data trends [37,38]. Other methods for modeling nonstationarities include autoregressive integrating moving averages (ARIMA) models [39] or regression-based forecasting models to extract complex characteristics of time series. More general models for state space representation also could be used to describe the motion of dynamic systems and extract position estimates as well as their derivatives eg, velocities or accelerations) from noisy data sources [40].

Regardless of the analytic approach, it may be that prediction of patients’ responses could be improved by including more prior information in the prediction (eg, information from a larger number of prior IVR assessments). In the current study, we attempted to strike a balance between maximizing the predictive accuracy for a given patient, and including in the analyses a large, more representative sample of patients with a sufficient number of assessments (ie, by requiring no more than five prior assessments with at least a 1-week, 2-week, and 1-month gap between each). Similar analyses in the context of data from large health plans may significantly improve the evidence base for clinical decision making.

### Conclusions

In summary, the content and frequency of current mobile health assessments is almost entirely based on a fixed schedule and expert opinion, rather than being individualized based on patients’ previously reported status. These analyses indicate that the technical feasibility of gathering high frequency health data may in some instances exceed the clinical benefit of doing so. In particular, weekly or biweekly depressive symptom reports may provide little marginal information regarding how the person is doing relative to collecting that information monthly. Data mining may allow us to detect trends in patient reports that can be used by intelligent systems to accurately predict patients’ health status. The next generation of automated health assessment services should use these or other data mining techniques to avoid redundant assessments and gather data at the frequency that maximizes the value of the information collected. Such adaptive systems could be much more patient-friendly and could accommodate a much broader set of risk factors for the large and growing number of patients who have multiple chronic diseases.

## Abbreviations

ANN: artificial neural networks
ARIMA: autoregressive integrating moving averages
AUC: area under the curve
IVR: interactive voice response calls
PHQ-9: 9-item Patient Health Questionnaire
ROC: Receiver Operator Characteristic curve
